# Mapping carbon utilization pathways in *Histoplasma capsulatum* through ^13^C-metabolic flux analysis

**DOI:** 10.1128/msystems.00569-25

**Published:** 2025-09-08

**Authors:** Adrian Heckart, Jean-Christophe Cocuron, Stephanie C. Ray, Gabriella F. Matheny, Chad A. Rappleye, Ana P. Alonso

**Affiliations:** 1 Department of Biological Sciences and BioDiscovery Institute, University of North Texas 465315, Denton, Texas, USA; 2 BioAnalytical Facility, University of North Texas 3404, Denton, Texas, USA; 3 Department of Microbiology, The Ohio State University 2647, Columbus, Ohio, USA; San Diego State University, San Diego, California, USA

**Keywords:** Metabolic flux analysis, fungal pathogen, isotopic labeling, biomass, Histoplasmosis, carbon flux map

## Abstract

*Histoplasma capsulatum* is a human fungal pathogen that survives and proliferates within phagocytic immune cells. To sustain growth in the nutrient-limited phagosome environment, the pathogenic yeast scavenges available carbon sources, which must be metabolized through central carbon metabolism for respiration and biomass synthesis. However, *Histoplasma* carbon metabolic pathways operating in the pathogenic yeast phase have not been extensively mapped. To address this gap, we employed a fluxomic platform using stable isotope tracers to quantify the cellular reaction rates of central carbon metabolism. This approach revealed that, in *Histoplasma* yeasts, carbon resides within five main reservoirs: fatty acids, proteins, mannitol, nucleic acids, and cell wall components. Carbon conversion efficiency, or biomass yield, was approximately 50%, indicating substantial CO_2_ loss from supplemented carbon substrates, glucose, and glutamate. ^13^C-labeling analysis demonstrated simultaneous glycolysis and gluconeogenesis, and enriched serine labeling confirmed threonine aldolase activity in serine biosynthesis. Compartmentalization of pyruvate metabolism was evident from the labeling of amino acids derived from pyruvate, with the methylcitrate cycle identified as the primary source of labeled pyruvate. Notably, malic enzyme and pyruvate carboxylase exhibited negligible fluxes, while mitochondrial reactions, particularly CO_2_-producing ones, were the most active. These results offer insight into key metabolic reactions, alternative pathways, and metabolite/enzyme compartmentalization in *Histoplasma* yeast metabolism. This foundational framework supports future studies aimed at identifying metabolic targets for novel histoplasmosis therapeutics.

**IMPORTANCE:**

To our knowledge, this study represents the first application of ^13^C-metabolic flux analysis to a human fungal pathogen, where we identified carbon reservoirs and quantified the metabolic fluxes of pathogenic *Histoplasma* yeasts. Our findings demonstrated that *Histoplasma* metabolizes carbon toward cellular respiration to robustly produce CO_2_ and energy but also uses alternative pathways within central metabolism for biosynthesis. Given the potential for other pathogenic fungi to share similar metabolic features, especially biomass, our study offers a comprehensive framework for deciphering fungal metabolism, providing insights into their infection-enabling metabolism and offering a foundation for identifying new therapeutic targets.

## INTRODUCTION

*Histoplasma capsulatum*, a human pathogen and the etiological agent of histoplasmosis, is responsible for the most common endemic mycosis in the United States (1–3). Human infection results from aerosolization and inhalation of conidia released from soil containing *Histoplasma* mycelia. Within the mammalian lung, conidia transition into pathogenic yeasts which then infect alveolar phagocytes (4, 5). Endemic to the Ohio and Mississippi River valleys, *Histoplasma* poses a significant threat to immunocompromised individuals, where infection can lead to severe respiratory disease and high mortality as the pathogen disseminates to other organs (6–8).

Despite *Histoplasma*’s significant impact, detailed insights into the central carbon metabolism of its pathogenic yeast phase and how it channels carbon through its metabolic pathways remain elusive. Genetic studies have shown that *Histoplasma* utilizes gluconeogenesis to process host-derived substrates within the nutrient-limited macrophage phagosome (9). More recent research revealed that *Histoplasma* is a Crabtree-negative yeast, favoring oxidative phosphorylation over alcoholic fermentation when both glucose and oxygen are available (10). Supporting these findings, a prior study demonstrated a reduction in enzymes associated with alcoholic fermentation and an increase in tricarboxylic acid (TCA) cycle enzymes during the transition from the mycelial to yeast form (11).

Additionally, whether alternative metabolic pathways in *Histoplasma*, including malic enzyme, the glyoxylate cycle, and the methylcitrate cycle, contribute to yeast phase metabolism has not been fully determined. These pathways have primarily been inferred through genome annotation and transcriptomic analyses rather than direct metabolic evidence, leaving their functional activity unresolved. For example, even though a malic enzyme ortholog (*MAE1*) has been annotated (12), previous evidence indicates that *Histoplasma* may lack malic enzyme function (9, 13). Likewise, glyoxylate and methylcitrate cycle enzymes were found to be downregulated two- to fourfold during its dimorphic shift from mycelia to yeasts (11), further questioning their activity, especially given that glyoxylate metabolism is not required for virulence (9). It also remains unclear if the methylcitrate cycle is required for virulence, possibly through catabolism of host-derived amino acids to propionyl-CoA, which may activate the methylcitrate cycle (14). These unresolved uncertainties in *Histoplasma*’s central carbon metabolism highlight the need for further investigation, as a clearer understanding could reveal potential metabolic vulnerabilities that may serve as targets for novel therapeutic interventions.

^13^C-metabolic flux analysis (MFA) offers a metabolic map that can be used to infer the role of different metabolic pathways (15–18). This technique relies on the incorporation of stable isotope-labeled substrates into a biological system. As the metabolites in the system achieve metabolic and isotopic steady state, where metabolite labeling and concentrations remain constant due to a balance between production and consumption, precise mass isotopomer distribution can be measured (19). These labeling patterns enable the calculation of metabolic fluxes, or reaction rates, by solving a system of equations that account for carbon inputs (e.g., substrates consumed), outputs (e.g., biomass accumulation and CO_2_ production), and carbon redistribution through metabolic reactions. Computer-aided modeling subsequently provides the best fit of the experimental isotope data with the predicted isotope data in conjunction with the built network to generate a flux map of central carbon metabolism (20).

Recent advancements in isotope tracing have made the measurement of intracellular metabolism rates an essential tool for developing novel therapies for some of the most concerning and prevalent diseases. For example, in breast cancer research, stable isotope modeling identified fluxes elevated by the Warburg effect (21), which were targeted by inhibitors, leading to tumor remission *in vitro* (22). Similarly, labeling of other cancer cell lines *in vitro* and *in vivo* identified a reductive TCA cycle driving fatty acid synthesis (23, 24). In *Mycobacterium tuberculosis*, ^13^C-flux spectral analysis, an approach akin to MFA, identified key amino acids accessible to the pathogen and highlighted CO_2_ fixation via anaplerotic reactions within macrophage phagosomes (25). These examples demonstrate the prospects of MFA to reveal metabolic vulnerabilities, paving the way for new therapeutic strategies across various diseases.

In this study, MFA was employed to investigate *Histoplasma*’s central carbon metabolism in its pathogenic yeast phase. By determining its biomass composition, rate of substrate uptake, and tracking the incorporation of ^13^C-labeled substrates into central metabolism, we generated a detailed carbon flux map of pathogenic-phase cells that identifies highly active reactions, reveals alternative metabolic pathways, and suggests possible metabolic compartmentalization within yeast cells.

## RESULTS

### *Histoplasma* respires half of all consumed carbon

**Fig 1:**
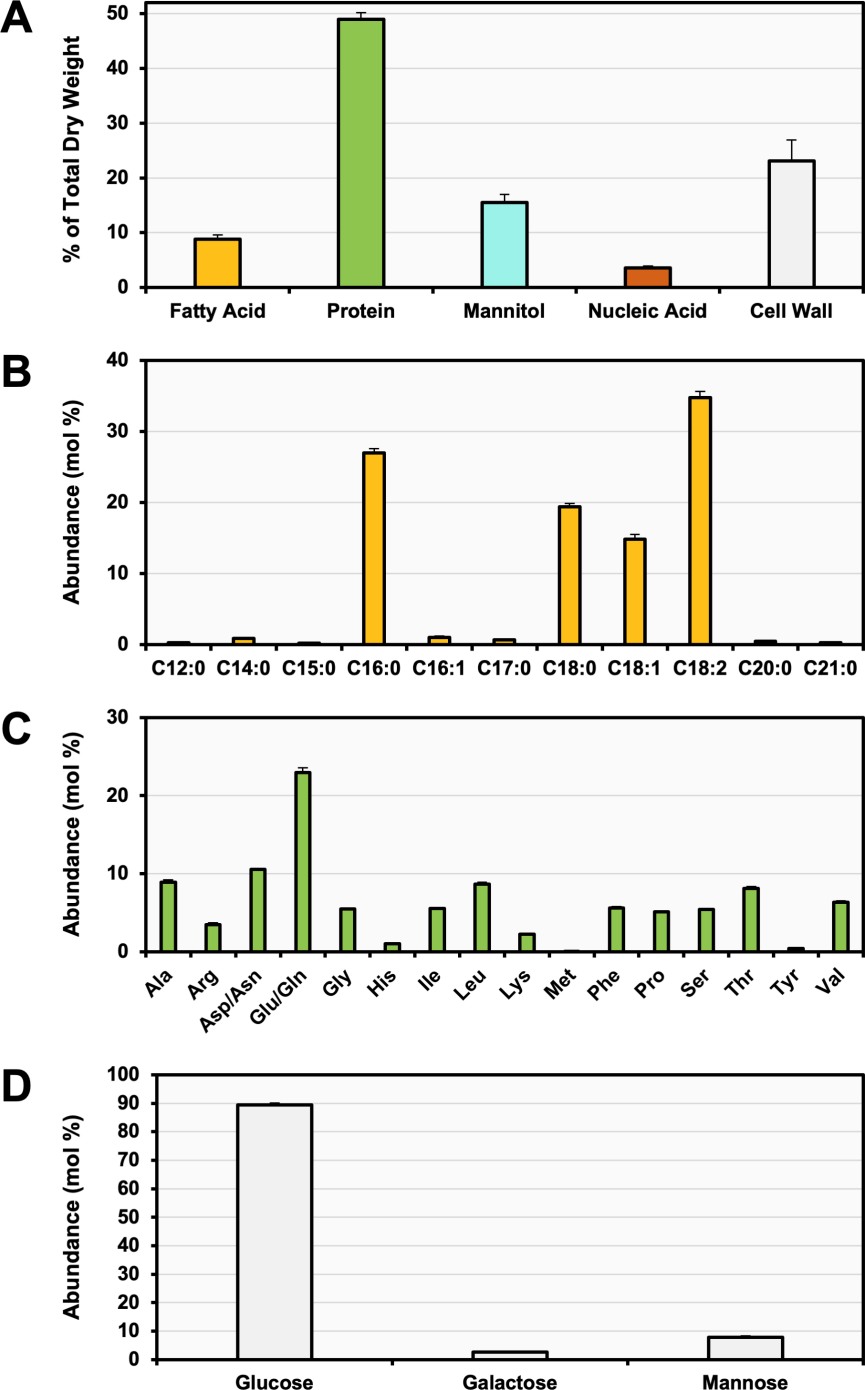
Biomass composition of *Histoplasma*. (**A**) Major biomass components include fatty acids (yellow), protein (green), mannitol (blue), and nucleic acid (brown), with remaining weight attributed to cell wall (light gray). Monomer composition is based on (**B**) acyl chains of the polar and nonpolar lipids, (**C**) proteinogenic amino acids, and (**D**) monosaccharides within the cell wall. Error bars represent standard deviations of five biological replicates. Cell wall error bars represent the sum of all standard deviations from other biomass components.

Fatty acids, proteins, nucleic acids, and carbohydrates are typically the primary reservoirs of carbon in yeasts (26–30). Accordingly, the biomass composition of *Histoplasma* yeast grown in liquid culture was assessed based on these components (Fig. 1A). For carbohydrates, previous research has shown *Histoplasma* yeasts produce the sugar alcohol mannitol in high abundance (10), but glycogen levels were under the limit of detection (data not shown; limit of detection = 0.18/100 g). Biomass analysis showed that fatty acids constituted 8.8% ± 0.8% of the total dry weight (DW; wt/wt), proteins 49.0% ± 1.2%, mannitol 15.5% ± 1.5%, and nucleic acids 3.6% ± 0.3%. The remaining DW was attributed to cell wall mass (23.1% ± 3.8%). Analysis of the fatty acyl chain distribution (Fig. 1B) revealed that palmitic acid (C16:0), stearic acid (C18:0), oleic acid (C18:1), and linoleic acid (C18:2) were the predominant fatty acids in *Histoplasma* yeasts, accounting for 96.0 mol% ± 2.5 mol% of the total fatty acids. Odd-chain fatty acids were also detected but were only 1.2 mol% ± 0.3 mol% of the total fatty acid types. The composition of proteinogenic amino acids (Fig. 1C) showed glutamate/glutamine as the most prevalent amino acids incorporated into proteins, constituting 23.0 mol% ± 0.6 mol%, with aspartate/asparagine and alanine following at 10.6 mol% ± 0.1 mol% and 8.9 mol% ± 0.3 mol%, respectively. Lastly, analysis of the monosaccharide composition of the cell wall (Fig. 1D) revealed that glucose, arising from glucans, and galactose and mannose, which can glycosylate cell wall proteins or together form galactomannan, were the only detectable carbohydrate monomers. Glucose was predominant, comprising 89.5 mol% ± 0.6 mol% of the monosaccharides in the cell wall, while mannose and galactose were present in a precise 3:1 ratio, accounting for 7.9 mol% ± 0.4 mol% and 2.6 mol% ± 0.1 mol%, respectively.

**Fig 2:**
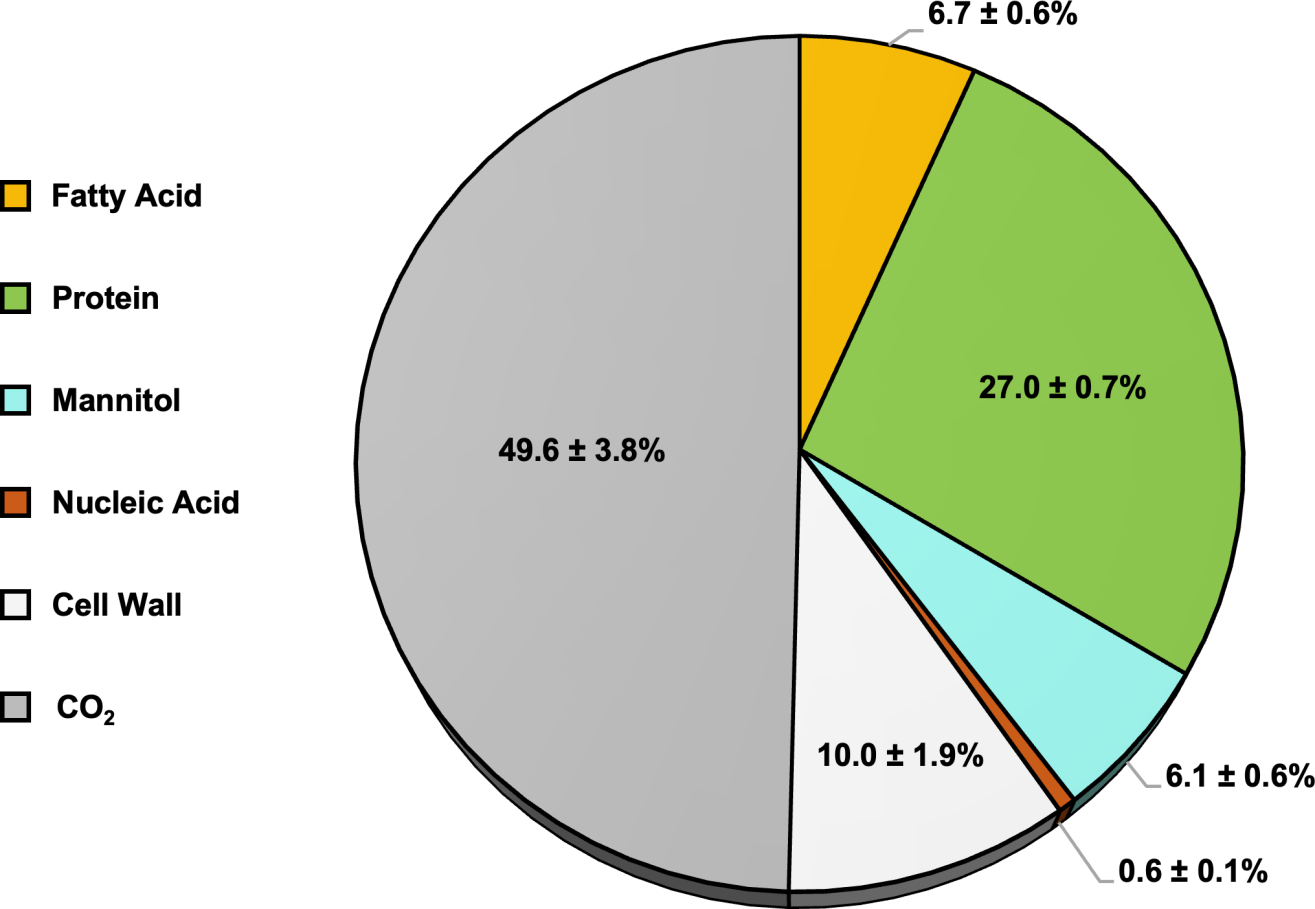
Half of the carbon uptaken by *Histoplasma* is lost as CO_2_. 50.4% of carbon consumed from the media is sunk into biomass components: fatty acids (yellow), protein (green), mannitol (blue), nucleic acid (brown), and cell wall (light gray). The remaining 49.6% of carbon consumed was attributed to CO_2_ release (dark gray). Values are the average of five biological replicates ± standard deviation. The standard deviation for the cell wall component includes the cumulative standard deviations from all other biomass fractions, and the CO_2_ standard deviation accounts for the summed standard deviations of the biomass components.

The efficiency of *Histoplasma* yeasts in converting substrates into biomass, also known as carbon conversion efficiency (CCE) or biomass yield, was determined by measuring the depletion of carbon substrates (glucose and glutamate) from rich growth medium over a 67 h incubation period and by quantifying the biomass components produced in µmol of carbon per mg DW (µmol C mg DW^−1^). The total carbon consumed from the medium was found to be 83.6 ± 17.8 µmol C mg DW^−1^, with 54.6 ± 13.7 µmol C mg DW^−1^ derived from glucose and 29.0 ± 4.1 µmol C mg DW^−1^ from glutamate. The total carbon stored in biomass components was 42.1 ± 3.2 µmol C mg DW^−1^, consisting of 5.6 ± 0.5, 22.6 ± 0.6, 5.1 ± 0.5, 0.5 ± 0.1, and 8.4 ± 1.6 µmol C mg DW^−1^ in lipids, proteins, mannitol, nucleic acids, and cell wall, respectively. Therefore, the CCE of *Histoplasma* was estimated at 50.4 ± 3.8% (Fig. 2), where 49.6 ± 3.8% of the carbon consumed by *Histoplasma* was lost as metabolic byproducts, presumably CO_2_ as yeasts do not ferment glucose to ethanol or acetate (10). Based on the CCE, central metabolic reactions such as 6-phosphogluconate dehydrogenase, pyruvate dehydrogenase, isocitrate dehydrogenase, and/or alpha-ketoglutarate dehydrogenase likely contain high flux to accommodate the production of CO_2_ measured.

### ^13^C-labeling of *Histoplasma* yeasts

Glucose and glutamate were selected as carbon substrates for *Histoplasma* growth to provide parallel ^13^C-labeling data within central metabolism (16, 17, 31–33). Specifically, 80% [1,2-^13^C_2_]glucose + 20% [U-^13^C_6_]glucose was expected to distinguish between glycolysis and the pentose phosphate pathway, while 100% [U-^13^C_5_]glutamate was intended to enrich intermediates of the TCA cycle and monitor potential gluconeogenesis (15–18, 34–36). The isotopic steady state of these metabolites was verified by incubating *Histoplasma* yeasts for 67 h with a 20% [U-^13^C_6_]glucose and 20% [U-^13^C_5_]glutamate mixture. Intracellular metabolites were extracted, and their isotopomer labeling was determined as outlined in the “Materials and Methods” section. For each metabolite, the average carbon labeling percentage was calculated as previously described (18), demonstrating that 34 out of the 47 analyzed metabolites reached isotopic steady state, with an average ^13^C-labeling between 18.0 and 24.0% (Table S1), similar to the labeling percentage of the carbon in the growth medium. Alanine, glutamine, glycine, lysine, methionine, and proline exhibited labeling percentages above the 24.0% threshold, likely due to interference from co-eluting compounds at the selected precursor/product ion transitions which increased their average carbon labeling percentages. Conversely, glutamate, 2-phosphoglycolate, glucose 1-phosphate/mannose 1-phosphate, alpha-ketoglutarate, succinate, glucose, and inositol were below the 18.0% threshold, likely due to dilution of the ^13^C-label by unlabeled compartmentalized and inactive pools of the respective metabolites. Due to these reasons, metabolites outside of the 18.0–24.0% threshold were excluded from further analyses.

Parallel labeling data from 29 of the 34 metabolites were included in the subsequent metabolic model from yeasts grown in the following media: 80% [1,2-^13^C_2_]glucose + 20% [U-^13^C_6_]glucose with unlabeled glutamate and unlabeled glucose with 100% [U-^13^C_5_]glutamate (Table S2). Asparagine and tryptophan were omitted from the model due to their absence from proteinogenic amino acid data, as they are degraded during the acid hydrolysis process used to break down proteins into their constituent amino acids (37). Mannose 6-phosphate, sorbitol 6-phosphate, and N-acetylglucosamine 1-phosphate were excluded due to redundancy in the model, as they did not contribute to any experimentally measured outputs as substrates.

**Fig 3:**
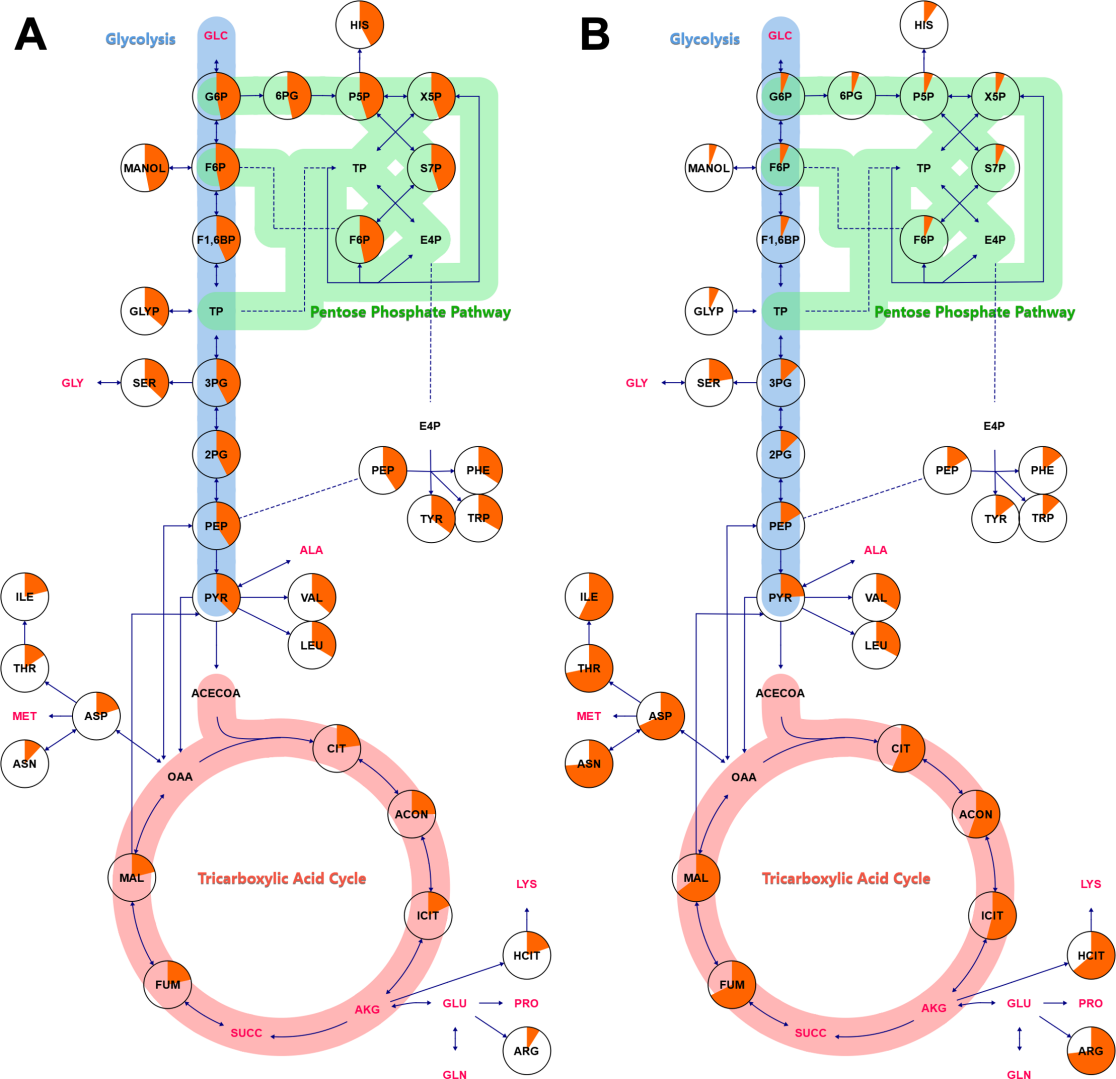
Metabolic maps illustrating simultaneous glycolysis and gluconeogenesis. The maps show metabolites labeled under two conditions: (**A**) 80% [1,2-^13^C_2_]glucose + 20% [U-^13^C_6_]glucose and (**B**) 100% [U-^13^C_5_]glutamate. The percent average labeling per carbon is represented by the orange fill percentage within each circle. Metabolites highlighted in red did not reach isotopic steady state after 67 h of incubation with 20% [U-^13^C_6_]glucose + 20% [U-^13^C_5_]glutamate. The average labeling percentages are based on five biological replicates, and isotopic steady-state determination is based on four biological replicates. GLC, glucose; G6P, glucose 6-phosphate; 6PG, 6-phosphogluconate; P5P, ribulose 5-phosphate/ribose 5-phosphate; HIS, histidine; X5P, xylulose 5-phosphate; TP, triose phosphate; S7P, sedoheptulose 7-phosphate; F6P, fructose 6-phosphate; E4P, erythrose 4-phosphate; PHE, phenylalanine; TYR, tyrosine; TRP, tryptophan; MANOL, mannitol; F1,6BP, fructose 1,6-bisphosphate; GLYP, glycerol phosphates; 3PG, 3-phosphoglycerate; SER, serine; GLY, glycine; 2PG, 2-phosphoglycerate; PEP, phosphoenolpyruvate; PYR, pyruvate; ALA, alanine; VAL, valine; LEU, leucine; ACECOA, acetyl-CoA; CIT, citrate; ACON, *cis*-aconitate; ICIT, isocitrate; AKG, alpha-ketoglutarate; HCIT, homocitrate; LYS, lysine; GLU, glutamate; GLN, glutamine; PRO, proline; ARG, arginine; SUCC, succinate; FUM, fumarate; MAL, malate; OAA, oxaloacetate; ASP, aspartate; THR, threonine; ILE, isoleucine; MET, methionine; ASN, asparagine.

To identify pathway trends with ^13^C-enrichment, the average carbon labeling percentages were mapped onto central metabolic pathways (Fig. 3). When ^13^C-glucose was the source of carbon label in the glucose and glutamate-containing growth medium (Fig. 3A), the most abundant labeling percentages in yeasts were predictably within intermediates in the upper portion of glycolysis. For instance, glucose 6-phosphate, the first product of glycolysis, showed a labeling percentage of 46.7% ± 0.2% while metabolites further away from the labeling entry point illustrated a steady decline in labeling percentages. Similarly, with ^13^C-glutamate in the growth medium (Fig. 3B), TCA intermediates had an average carbon labeling of 54.7–67.2%, while label of intermediates of upper glycolysis and the pentose phosphate pathway ranged from 3.4 to 6.6% (Table S2), significantly higher than the natural abundance of ^13^C at 1.1%. As both labeling experiments were performed using media with both glucose and glutamate carbon sources, these observations indicate that both glycolysis and gluconeogenesis pathways are simultaneously active in *Histoplasma*. Additionally, serine’s ^13^C-labeling with glutamate averaged 22.3% ± 4.7%, whereas its direct precursor, 3-phosphoglycerate, was lower (13.0% ± 1.2%; Table S2), suggesting an alternative serine biosynthesis pathway possibly via threonine aldolase (38). Lastly, valine and leucine, synthesized exclusively from mitochondrial pyruvate (39, 40), exhibited 9.7% higher labeling than the measured total pyruvate pool (Fig. 3B). This discrepancy supports the existence of compartmentalized pyruvate pools, where mitochondrial pyruvate is generated independently of the cytosolic pool. With growth on ^13^C-glutamate, increased labeling of branched-chain amino acids could result from mitochondrial pyruvate production via either mitochondrial malic enzyme or the methylcitrate cycle, which metabolizes propionyl-CoA biosynthesized from 2-oxobutanoate, where 2-oxobutanoate is derived from oxaloacetate. In contrast, the lack of differences in pyruvate and valine/leucine carbon labeling with ^13^C-glucose, where labeled pyruvate would be derived from glycolysis, suggests that the cytosolic pyruvate pool is larger and not significantly diluted by the mitochondrial pyruvate fraction.

### Metabolic modeling and validation

A metabolic model (Table S3) was built using the isotopomer network compartmental analysis (INCA v2.3, http://mfa.vueinnovations.com, Vanderbilt University) (20) within MATLAB 2023b (MathWorks, Natick, MA, USA) using information from the Kyoto Encyclopedia of Genes and Genomes (38), current literature on the enzymes and metabolites produced by *Histoplasma* (10–12, 41), and the labeling data obtained in this study (Table S2). The network of carbon atom transitions was adapted from one previously published (18). Regarding directionality, *Histoplasma* performs both glycolysis and gluconeogenesis simultaneously (Fig. 3). Therefore, Vhk and Vpfk were modeled as bidirectional to simulate the enzymes catalyzing the reverse reactions: glucose 6-phosphatase and fructose 1,6-bisphosphatase, respectively. This is further supported by the observed isotopic scrambling, where labeling with ^13^C-glucose results in M + 3 isotopologues in hexose phosphates due to aldolase and gluconeogenic reactions (35). Reactions involved in central metabolism that are known to be thermodynamically unfavorable, including Vpk, Vme, Vpyrc, Vpyr, Vpdh, Vcs, Vatpcl, Vkgdh, Vg6pdh, V6pgdh, Vms, Voxo, Vmetcit, Vimetcit, and Vmicl, were implemented as unidirectional in INCA according to their conventional directionality. However, isocitrate dehydrogenase (Vidh) was maintained as bidirectional due to increasing evidence of its reversibility in various organisms (34, 35, 42, 43). Regarding compartmentalization, the model also incorporated separate pools of pyruvate, with mitochondrial pyruvate allocated for branched-chain amino acid biosynthesis and cytosolic pyruvate for alanine biosynthesis. The measured mass isotopomer distribution of pyruvate (Table S2) was assigned to the cytosolic pool due to its inferred larger relative abundance. Glyoxylate, malic enzyme, and methylcitrate cycle reactions were included based on the detection of their enzymes and intermediates in the literature (10, 11, 41), and for the latter two, the increased labeling observed in branched-chain amino acids derived from mitochondrial pyruvate (Fig. 3B). Similarly, threonine aldolase was added to the model, supported by the observed average carbon labeling of serine (22.3% ± 4.7%) compared to its precursor, 3-phosphoglycerate (13.0% ± 1.2%; Table S2), indicating an alternative serine biosynthesis pathway.

**Fig 4:**
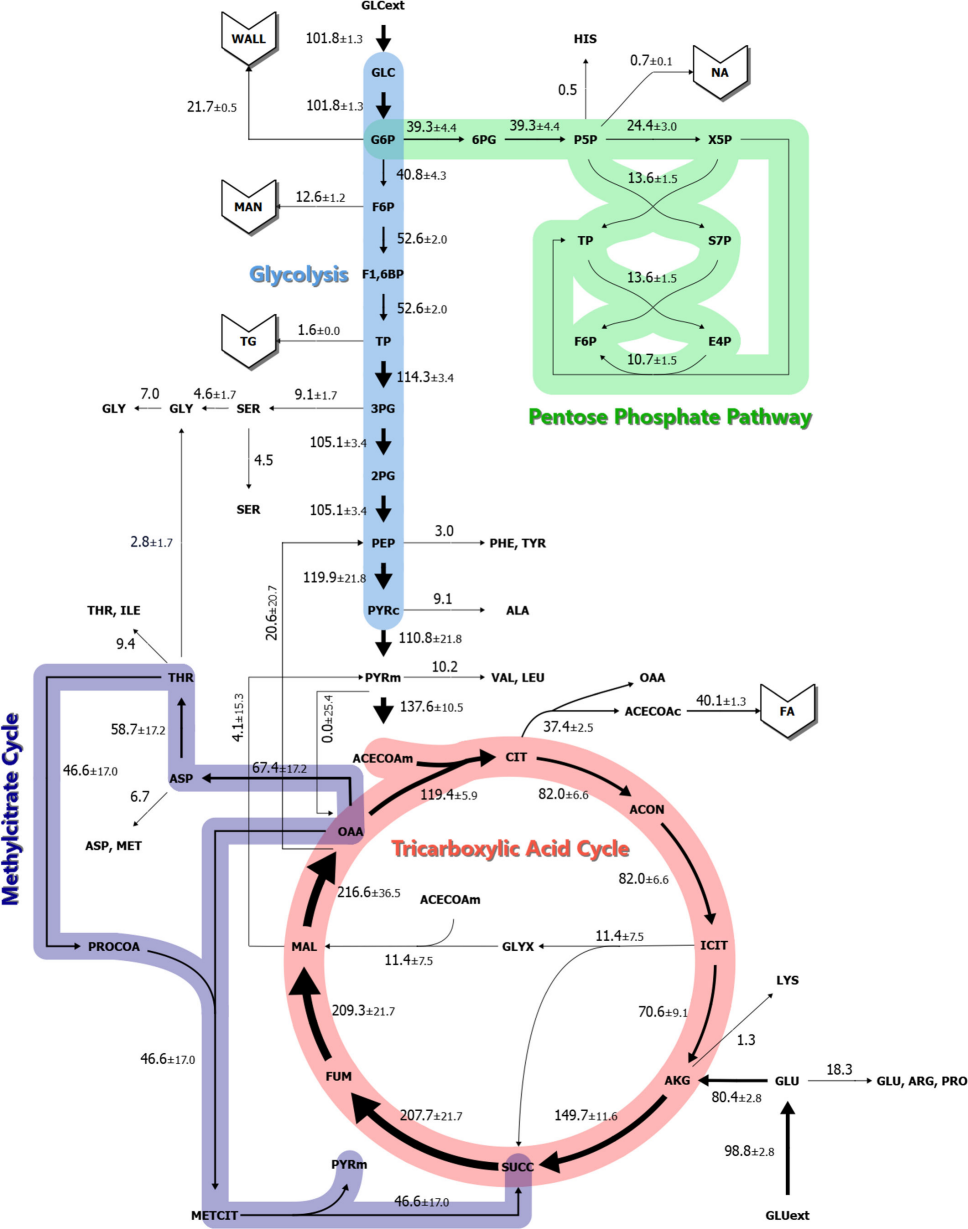
Metabolic network and fluxes in *Histoplasma*. The map illustrates the central carbon metabolism, showing the net calculated fluxes determined using INCA software after labeling with 80% [1,2-^13^C_2_]glucose + 20% [U-^13^C_6_]glucose and 100% [U-^13^C_5_]glutamate. Flux values are presented in nmol h^−1^ mg DW^−1^ with the confidence interval. Arrow widths are proportional to the net flux values. Ext, extracellular; c, cytosolic; m, mitochondrial; GLC, glucose; G6P, glucose 6-phosphate; WALL, cell wall; 6PG, 6-phosphogluconate; P5P, ribulose 5-phosphate/ribose 5-phosphate; HIS, histidine; NA, nucleic acids; X5P, xylulose 5-phosphate; TP, triose phosphate; S7P, sedoheptulose 7-phosphate; F6P, fructose 6-phosphate; E4P, erythrose 4-phosphate; MAN, mannitol; F1,6BP, fructose 1,6-bisphosphate; TG, triacylglycerol; 3PG, 3-phosphoglycerate; SER, serine; GLY, glycine; 2PG, 2-phosphoglycerate; PEP, phosphoenolpyruvate; PHE, phenylalanine; TYR, tyrosine; PYR, pyruvate; ALA, alanine; VAL, valine; LEU, leucine; ACECOA, acetyl-CoA; CIT, citrate; OAA, oxaloacetate; FA, fatty acid; ACON, cis-aconitate; ICIT, isocitrate; GLYX, glyoxylate; AKG, alpha-ketoglutarate; LYS, lysine; GLU, glutamate; PRO, proline; ARG, arginine; SUCC, succinate; FUM, fumarate; MAL, malate; ASP, aspartate; MET, methionine; THR, threonine; ILE, isoleucine; PROCOA, propionyl-CoA; METCIT, methylcitrate.

The model’s calculated net and exchange fluxes are presented in Table 1, with net fluxes visualized in Fig. 4. The consistency between the experimental and model-predicted labeling patterns (Fig. S1) was used to assess the goodness of fit, using methods previously outlined (16–18). Plotting of the experimental mass isotopomer distributions versus the model-predicted mass isotopomer distributions for each measured metabolite provided a means to evaluate the model’s accuracy. The resulting linear regression showed a slope close to one and a coefficient of determination (*R*^2^) near 1, indicating strong agreement between the simulated and observed labeling data and supporting the model’s ability to accurately represent in culture fluxes in *Histoplasma*. To account for the ambiguous subcellular localizations of pyruvate carboxylase (44), malate synthase, and methylisocitrate lyase in fungi (45), flux estimation was repeated using multiple localization assignments for acetyl-CoA and pyruvate, aiming to minimize residuals between predicted and observed isotopologues. Residuals were minimized by these adjustments (data not shown), and in the revised model, pyruvate carboxylase produced a net flux of less than 0.1 regardless of localization. Conversely, malate synthase, and methylisocitrate lyase resulted in the lowest residuals when assigned to mitochondrial localization.

**TABLE 1:** Net and exchange carbon fluxes in *Histoplasma^a^*

Flux name	Flux description	Flux values (nmol h^−1^ mg DW^−1^)	
		Net flux (CI)	Exchange flux (CI)
Vglcup	Flux of glucose uptake	101.8 (101.8–104.4)	0 (0)
Vgluup	Flux of glutamate uptake	98.8 (93.2–98.8)	0 (0)
Vfas1	Flux of glycerol incorporation into triacylglycerols	1.6 (1.5–1.6)	0 (0)
Vfas2	Flux of cytosolic acetyl-CoA into triacylglycerols	40.1 (40.1–42.7)	0 (0)
Vmaneff	Flux of mannitol synthesis	12.6 (11.4–13.9)	0 (0)
Vpurine	Flux of purine synthesis	0.4 (0.4–0.5)	0 (0)
Vpyrim	Flux of pyrimidine synthesis	0.4 (0.4–0.5)	0 (0)
Vwall	Flux of cell wall component synthesis	21.7 (20.6–21.7)	0 (0)
Valaeff	Flux of alanine used for protein synthesis	9.1	0 (0)
Vargeff	Flux of arginine used for protein synthesis	1.6	0 (0)
Vaspeff	Flux of aspartate used for protein synthesis	6.7	0 (0)
Vglueff	Flux of glutamate used for protein synthesis	12.9	0 (0)
Vglyeff	Flux of glycine used for protein synthesis	7.0	0 (0)
Vhiseff	Flux of histidine used for protein synthesis	0.5	0 (0)
Vileeff	Flux of isoleucine used for protein synthesis	3.6	0 (0)
Vleueff	Flux of leucine used for protein synthesis	5.6	0 (0)
Vlyseff	Flux of lysine used for protein synthesis	1.3	0 (0)
Vmeteff	Flux of methionine used for protein synthesis	0.0	0 (0)
Vpheeff	Flux of phenylalanine used for protein synthesis	2.8	0 (0)
Vproeff	Flux of proline used for protein synthesis	3.8	0 (0)
Vsereff	Flux of serine used for protein synthesis	4.5	0 (0)
Vthreff	Flux of threonine used for protein synthesis	5.8	0 (0)
Vtyreff	Flux of tyrosine used for protein synthesis	0.2	0 (0)
Vvaleff	Flux of valine used for protein synthesis	4.6	0 (0)
Vch4eff	Flux of the single carbon transferable unit in biotin	3.3 (2.0–5.5)	0 (0)
Vco2	Flux of carbon dioxide production	490 (449.9–498.2)	0 (0)
Vala	Flux of alanine synthesis	9.1 (9.1–9.1)	0 (0.0–0.1)
Varg	Flux of arginine synthesis	1.6 (1.6–1.6)	10,000,000 (818.2–∞)
Vasp	Flux of aspartate synthesis	67.4 (46.6–80.9)	0 (0.0–107.2)
Vgly1	Flux of glycine synthesis from serine	4.6 (3.4–6.9)	21.7 (14.4–36.8)
Vgly2	Flux of glycine synthesis from threonine	2.8 (0.5–4.0)	0 (0.0–2.7)
Vhis	Flux of histidine synthesis	0.5 (0.5–0.5)	0 (0)
Vile	Flux of isoleucine synthesis	3.6 (3.6–3.6)	0 (0)
Vleu	Flux of leucine synthesis	5.6 (5.6–5.6)	0 (0)
Vlys1	Flux of homocitrate synthesis	1.3 (1.3–1.3)	0 (0)
Vlys2	Flux of lysine synthesis from homocitrate	1.3 (1.3–1.3)	0 (0)
Vmet	Flux of methionine synthesis	0 (0.0–0.0)	0 (0)
Vphe	Flux of phenylalanine synthesis	2.8 (2.8–2.8)	0 (0)
Vpro	Flux of proline synthesis	3.8 (3.8–3.8)	0 (0)
Vser	Flux of serine synthesis	9.1 (7.9–11.4)	0 (0)
Vthr	Flux of threonine synthesis	58.7 (37.9–72.2)	0 (0)
Vtyr	Flux of tyrosine synthesis	0.2 (0.2–0.2)	0 (0)
Vval	Flux of valine synthesis	10.2 (10.2–10.2)	0 (0)
Vald	Flux catalyzed by aldolase	52.6 (50.6–54.5)	564.9 (337.4–1,350.3)
Veno	Flux catalyzed by enolase	105.1 (101.1–108.0)	169.2 (127.6–257.3)
Vglyp	Flux of glycerol phosphate synthesis	1.6 (1.5–1.6)	0.1 (0.0–0.2)
Vhk	Flux catalyzed by hexokinase	101.8 (101.8–104.4)	4,255.7 (0.0–6,818.3)
Vman	Flux of mannitol synthesis	12.6 (11.4–13.9)	219 (143.4–330.8)
Vpfk	Flux catalyzed by phosphofructokinase	52.6 (50.6–54.5)	80.4 (66.1–96.9)
Vpgi	Flux catalyzed by phosphoglucose isomerase	40.8 (37.3–45.9)	10,000,000 (6,823,800.0–∞)
Vpgk	Flux catalyzed by phosphoglycerate kinase	114.3 (110.9–117.7)	244.3 (158.9–545.9)
Vpgm	Flux catalyzed by phosphoglycerate mutase	105.1 (101.1–108.0)	8,470,600 (484.9–∞)
Vpk	Flux catalyzed by pyruvate kinase	119.9 (109.8–153.3)	0 (0)
Vme	Flux catalyzed by malic enzyme	4.1 (0.0–30.7)	0 (0)
Vpepc	Flux catalyzed by phosphoenolpyruvate carboxykinase	20.6 (12.9–54.4)	43.2 (8.0–51.3)
Vpyrc	Flux catalyzed by pyruvate carboxylase	0 (0.0–50.8)	0 (0)
Vaco1	Flux catalyzed by aconitase between citrate and aconitate	82 (71.1–84.3)	4,916,700 (175.5–∞)
Vaco2	Flux catalyzed by aconitase between aconitate and isocitrate	82 (71.1–84.3)	447.9 (139.3–∞)
Vatpcl	Flux catalyzed by adenosine triphosphate citrate lyase	37.4 (36.3–41.3)	0 (0)
Vcs	Flux catalyzed by citrate synthase	119.4 (110.8–122.6)	0 (0)
Vfum	Flux catalyzed by fumarase	209.3 (178.8–222.2)	0 (0.0–135.8)
Vgdh	Flux catalyzed by glutamate dehydrogenase	80.4 (74.8–80.4)	1,165.6 (452.1–∞)
Vidh	Flux catalyzed by isocitrate dehydrogenase	70.6 (58.4–76.6)	14 (6.2–18.5)
Vkgdh	Flux catalyzed by alpha-ketoglutarate dehydrogenase	149.7 (132.6–155.7)	0 (0)
Vmdh	Flux catalyzed by malate dehydrogenase	216.6 (161.6–234.5)	9,999,800 (∞–∞)
Vpdh	Flux catalyzed by pyruvate dehydrogenase	137.6 (125.0–146.0)	0 (0)
Vpyr	Flux of pyruvate from cytosol to mitochondria	110.8 (100.7–144.2)	0 (0)
Vsdh	Flux catalyzed by succinate dehydrogenase	207.7 (177.2–220.6)	16.5 (0–∞)
V6pgdh	Flux catalyzed by 6-phosphogluconate dehydrogenase	39.3 (34.5–43.4)	0 (0)
Vg6pdh	Flux catalyzed by glucose 6-phosphate dehydrogenase	39.3 (34.5–43.4)	0 (0)
Vr5pe	Flux catalyzed by ribulose 5-phosphate epimerase	24.4 (21.1–27.0)	422.7 (39.7–∞)
Vta	Flux catalyzed by transaldolase	13.6 (12.0–15.0)	0.1 (0.0–5.4)
Vtk1	Flux catalyzed by transketolase (P5P, X5P, TP, S7P)	13.6 (12.0–15.0)	50.4 (31.7–76.1)
Vtk2	Flux catalyzed by transketolase (X5P, E4P, F6P, TP)	10.7 (9.1–12.0)	19.1 (14.2–25.3)
Vicl	Flux catalyzed by isocitrate lyase	11.4 (3.3–18.3)	9,418,200 (422.5–∞)
Vms	Flux catalyzed by malate synthase	11.4 (3.3–18.3)	0 (0)
Vimetcit	Flux of isomethylcitrate synthesis	46.6 (26.2–60.2)	0 (0)
Vmetcit	Flux of methylcitrate synthesis	46.6 (26.2–60.2)	0 (0)
Vmicl	Flux catalyzed by methylisocitrate lyase	46.6 (26.2–60.2)	0 (0)
Voxo	Flux of 2-oxobutanoate synthesis	50.1 (29.8–63.8)	0 (0)
Vprocoa	Flux of propionyl-CoA synthesis	46.6 (26.2–60.2)	0 (0)

^*a*^ Flux values, expressed in nmol h^−1^ mg DW^−1^, were calculated using INCA software. Vglcup and Vgluup were determined by LC-MS/MS following 67 h of *istoplasma* incubation. These rates, along with their standard deviations, were treated as free fluxes and fitted using the flux estimation tool. Vwall, Vfas1, Vfas2, Vmaneff, Vpyrim, Vpurine, and all amino acid effluxes were quantified based on DW and biomass accumulation after 67 h. Proteinogenic amino acid effluxes were set as constrained values in the model. All other fluxes were derived from the model based on ^13^C-labeling quantification after 67 h incubation with 80% [1,2-^13^C_2_]glucose + 20% [U-^13^C_6_]glucose and 100% [U-^13^C_5_]glutamate. Values represent the best-fit optimized flux means with their 95% confidence intervals (CI).

Analysis of the flux estimation results revealed that all reactions operate with positive net fluxes in their conventional forward direction (Fig. 4). Excluding biomass-producing reactions, the largest fluxes in *Histoplasma*’s central metabolism were associated with the TCA cycle. Notably, malate dehydrogenase catalyzed the highest flux (216.6 ± 36.5 nmol h^−1^ mg DW^−1^), followed closely by fumarase (209.3 ± 21.7 nmol h^−1^ mg DW^−1^) and succinate dehydrogenase (207.7 ± 21.3 nmol h^−1^ mg DW^−1^). Alpha-ketoglutarate dehydrogenase and pyruvate dehydrogenase also exhibited substantial fluxes of 149.7 ± 11.6 and 137.6 ± 10.5 nmol h^−1^ mg DW^−1^, respectively. Oppositely, the smallest fluxes in *Histoplasma*’s central metabolism included pyruvate carboxylase (<0.1 ± 25.4 nmol h^−1^ mg DW^−1^), malic enzyme (4.1 ± 15.3 nmol h^−1^ mg DW^−1^), all nonoxidative pentose phosphate pathway reactions (10.7−13.6 ± 1.5 nmol h^−1^ mg DW^−1^), and glyoxylate cycle reactions (11.4 ± 7.5 nmol h^−1^ mg DW^−1^).

As half of the carbon uptaken by *Histoplasma* was lost as CO_2_ (Fig. 2), CO_2_ output by CO_2_-producing reactions was analyzed, where mitochondrial fluxes similarly represented the largest contributions. Specifically, 72.7% of total CO_2_ yield occurred through alpha-ketoglutarate dehydrogenase, pyruvate dehydrogenase, and isocitrate dehydrogenase (Table 1). An additional 9.5% of CO_2_ was generated during the conversion of 2-oxobutanoate to propionyl-CoA via the branched-chain-keto acid dehydrogenase complex located within the mitochondrial inner membrane (46). The fifth largest CO_2_-producing flux was attributed to 6-phosphogluconate dehydrogenase in the oxidative pentose phosphate pathway, accounting for 8.0% of total CO_2_ production.

## DISCUSSION

This study explored the central carbon metabolism of *Histoplasma* in its pathogenic yeast phase. By quantifying the distribution of major biomass components and analyzing their carbon precursors, a profile of *Histoplasma*’s substrate utilization and carbon allocation was generated. Subsequent ^13^C-labeling experiments supported these carbon contributions and identified active metabolic pathways and potential compartmentalization of metabolic reactions. Altogether, the analysis of labeling patterns and flux values revealed key features of *Histoplasma*’s central metabolism, including highly active and minimally utilized carbon pathways, alternative metabolic routes, and evidence of compartmentalized metabolism. We acknowledge that a limitation of this study is the use of *Histoplasma* yeasts grown in liquid culture, chosen due to the feasibility of MFA in defined media. While the nutrient environment may differ from that within host macrophages, we incorporated both glucose and glutamate in the medium and performed ^13^C-labeling experiments to assess major carbon entry points. Several flux patterns observed are consistent with previous findings related to intracellular yeast metabolism, indicating the relevance of results from *in vitro* culture. For example, flux analysis showed minimal activity of the glyoxylate cycle consistent with its dispensability for intracellular growth (9). The activity of gluconeogenesis, even when ample glucose is available in the growth medium, is also consistent with findings that gluconeogenesis is necessary for intracellular growth of *Histoplasma* yeasts. Finally, the finding that the methylcitrate cycle is active suggests its importance in catabolism of some amino acids (see below) rather than odd-chain fatty acids, which were largely absent (Fig. 1B), aligning with implications of recent studies on intracellular metabolism (9, 10, 47, 48). Thus, the findings of our study, particularly the flux defined by ^13^C-glutamate labeling of yeasts grown *in vitro*, provide an important framework on which many pathways relevant to intracellular yeasts are found.

### Carbon utilization for energy versus biomass in *Histoplasma*

Biomass yield (CCE) in *Histoplasma* yeast growth exhibited a CCE of 50.4% ± 3.8% when grown aerobically on glucose and glutamate, consistent with other yeast species that show CCE yields between 40 and 60% (29, 49–51). Since *Histoplasma* is an obligate aerobe (52) and ethanol was not detected under normoxic conditions (10), the observed high flux through mitochondrial reactions, especially CO_2_ production, was expected (Fig. 4). Oppositely, we found a lack of flux through pyruvate carboxylase activity despite detection of pyruvate carboxylase in proteomic studies (53–55) and RNA-sequencing data (12). This enzyme, a key contributor to anaplerosis by replenishing TCA cycle intermediates during gluconeogenesis, showed zero flux in the model regardless of modeling its localization to the cytosol or mitochondria. One possible explanation for the discrepancy is that pyruvate carboxylase activity was allosterically inhibited by glutamate (56–58), which accumulated in *Histoplasma* under carbon-rich conditions (10). Alternatively, because the cited proteomic studies were performed on secreted vesicles, they may not accurately reflect the intracellular proteome. It is also possible that *Histoplasma* pyruvate carboxylase has intrinsically low enzymatic activity.

### Pyruvate is generated through multiple pathways

**Fig 5:**
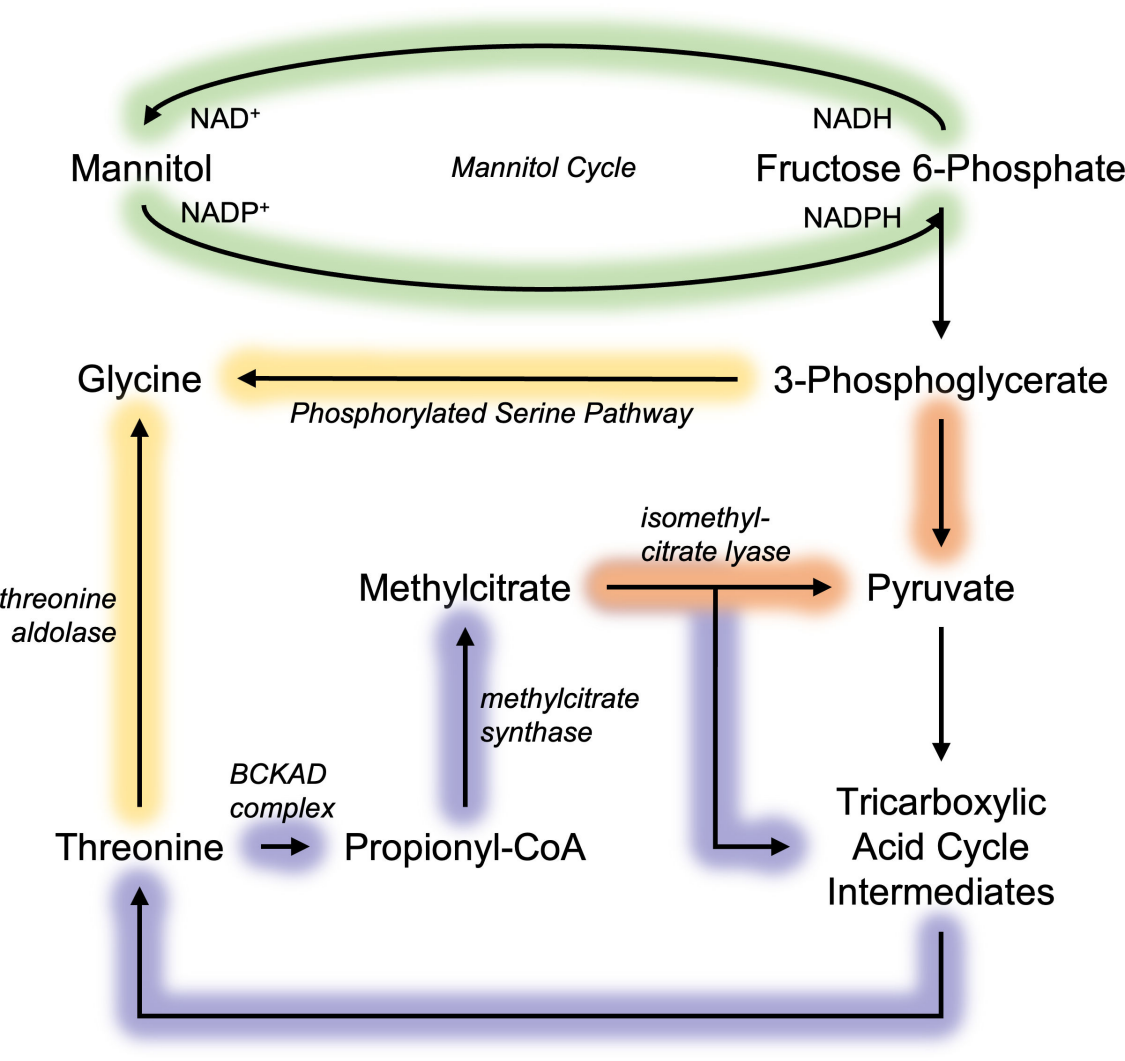
Summary schematic of metabolic pathways in *Histoplasma*. Arrows highlighted in orange represent reactions contributing to pyruvate production, in purple represent the methylcitrate cycle, in green represent the mannitol cycle, and in yellow represent reactions contributing to glycine production.

The ^13^C-labeling patterns of sugar phosphates when yeasts were cultured with ^13^C-glutamate were significantly higher than the natural ^13^C abundance (1.1%) (Fig. 3B), indicating active gluconeogenesis and corroborating prior genetic studies in *Histoplasma* (9). This enrichment predominantly occurred via phosphoenolpyruvate carboxykinase, which converted M + 4 oxaloacetate to M + 3 phosphoenolpyruvate (Table S2). However, the M + 3 isotopomer abundance of phosphoenolpyruvate (10.8%) was lower than that of pyruvate (18.6%), suggesting that alternative pathways contribute to pyruvate synthesis than from glycolysis (Fig. 5). Potential avenues for pyruvate production include the mitochondrial malic enzyme (decarboxylation of malate to pyruvate) or methylisocitrate lyase (part of the methylcitrate cycle for metabolism of propionyl-CoA from amino acids and beta-oxidation of odd-chain fatty acids). MFA resolved these two possibilities, revealing that malic enzyme plays a minimal role in mitochondrial pyruvate production, whereas methylisocitrate lyase accounted for over a quarter of its synthesis (Fig. 4). These findings highlight the active role of the methylcitrate cycle in *Histoplasma*’s central metabolism and align with earlier findings of inactive malic enzyme activity in pyruvate kinase-deficient yeasts (9). Furthermore, this indicates that mitochondrial pyruvate is separate from cytosolic pyruvate pools and suggests that methylisocitrate lyase may be compartmentalized in the mitochondria. Had either of these mechanisms been functional in the cytosol, they would have at least partially compensated for pyruvate kinase deficiency that led to abolished growth under gluconeogenic amino acids, as previously reported (9).

### The potential of methylcitrate and mannitol cycles in production of reducing power

The methylcitrate cycle appears to play a broader role in mitochondrial metabolism beyond its traditional function in detoxifying propionate derived from amino acid and odd-chain fatty acid catabolism (59, 60). We hypothesize that this pathway may compensate for lack of malic enzyme activity to produce pyruvate and additionally synthesize succinate without the carbon losses associated with isocitrate dehydrogenase or alpha-ketoglutarate dehydrogenase. For instance, the branched-chain alpha-keto acid dehydrogenase complex decarboxylates 2-oxobutanoate (with carbon ultimately derived from oxaloacetate) to form propionyl-CoA, producing CO_2_ and NADH reductant (Fig. 5; 61). Given that isoleucine and valine are synthesized from mitochondrial pyruvate and odd-chain fatty acids represent a minimal fraction of the fatty acid pool (Fig. 1B), these sources are unlikely to drive substantial flux through the methylcitrate cycle. In the nutrient-limited environment of the macrophage phagosome, where oxaloacetate-derived amino acids may be utilized toward gluconeogenesis during infection (9), the methylcitrate cycle may instead promote carbon conservation and reductant regeneration, supporting *Histoplasma* pathogenesis.

Generating NADPH is essential for biosynthesis and is also one output of the mitochondrial malic enzyme, which appears minimally active in *Histoplasma* yeasts (Fig. 4). Since the methylcitrate cycle cannot compensate for this function of malic enzyme, NADPH biosynthesis may rely on the mannitol cycle, which consumes NADH for mannitol synthesis and its catabolism generates NADPH in a cyclical manner (Fig. 5; 62). Interestingly, mannitol reached isotopic steady state despite its vast pool size (Table S1) (10), unlike glutamate, which was diluted. This suggests that mannitol undergo active turnover rather than serve as a stagnant carbon pool, consistent with metabolic network principles (19). Thus, in the absence of malic enzyme for NADPH homeostasis, the mannitol cycle may serve a critical role in NADPH production in exchange for NADH in *Histoplasma*’s metabolism, as observed in other fungi (63–65).

Given the importance of mannitol in NADPH homeostasis to serving as a carbohydrate store and possibly detoxifying the phagosomal space of reactive oxygen species (10), disrupting these functions could lead to novel therapeutics. Nitrophenide has been reported as an inhibitor of mannitol metabolism in the filamentous fungus *Neosartorya fischeri* (66). While there are notable differences in morphotypes between *Histoplasma* and filamentous fungi and the compound remains uncharacterized in pathogenic yeasts, its potential as an inhibitor in *Histoplasma* warrants consideration. Further investigation is needed to assess its relevance and efficacy.

### Dual pathways of serine biosynthesis in *Histoplasma* yeasts

Other alternative routes of carbon metabolism were also suggested by ^13^C-labeling flux analysis. Within yeasts labeled with ^13^C-glutamate (Fig. 3B), serine notably had an average labeling percent dissimilar to its direct precursor, 3-phosphoglycerate, while matching average labeling to pyruvate. However, the mass isotopomer distribution of serine differed significantly from that of pyruvate, with serine showing an abundance of M + 1 and M + 2 isotopomers, while pyruvate was dominated by M + 3 isotopomers (Table S2). These results indicate that serine is neither substantially deaminated to pyruvate nor reconverted into 3-phosphoglycerate (via reversible phosphorylated serine pathway), as such processes would have been reflected by similar isotopomer profiles. Instead, serine biosynthesis may proceed via an alternative pathway involving threonine aldolase, which generates glycine, followed by its reversible conversion of glycine into serine via glycine hydroxymethyltransferase (Fig. 5; 38). Transcripts for these two enzymes have been detected in prior studies utilizing similar growth conditions (accession #: PRJNA1069744). These findings demonstrate that *Histoplasma* produces serine through two distinct, concurrently active metabolic pathways, rather than relying solely on synthesis from 3-phosphoglycerate.

In conclusion, this study reveals the central carbon metabolism of pathogenic phase yeast cells of *Histoplasma*, providing new insights into its major carbon sinks, active metabolic reactions and pathways, and enzyme compartmentalization. This study also showcases the potential of fluxomics in analyzing the activity of enzymatic reactions and that active metabolic pathways cannot be assumed simply from genes encoding pathway enzymes or the mere availability of pathway substrates. To this end, comparative MFA between strains, mutants, morphotypes, and other fungal pathogens offers a promising path forward for uncovering metabolism that facilitates pathogenesis.

## MATERIALS AND METHODS

### Chemicals

Fatty acids were extracted and quantified using glyceryl trinonadecanoate (TG-C19:0), methyl tert-butyl ether, toluene, dichloromethane, sodium bisulfate, and sulfuric acid (MilliporeSigma, St. Louis, MO, USA). Glycogen was extracted and quantified with glacial acetic acid (MilliporeSigma), glycogen standard (Thermo Fisher Scientific, Waltham, MA, USA), and a total starch assay kit (Megazyme, Bray, IE). Nucleic acid extraction utilized Triton X-100 (MilliporeSigma), NaCl and tris(hydroxymethyl)aminomethane (Tris) (Research Products International, Mt. Prospect, IL, USA), ethylenediaminetetraacetic acid (EDTA) (Invitrogen, Carlsbad, CA, USA), phenol and isoamyl alcohol (Thermo Fisher Scientific), ethanol (Decon Labs, Inc, King of Prussia, PA, USA), and sodium acetate (J. T. Baker, Inc, Phillipsburg, NJ, USA). Cell walls were extracted with acetone, ethyl acetate, and trifluoracetic acid (Thermo Fisher Scientific) and with acetic anhydride, ammonium hydroxide, pyridine, and sodium borohydride (MilliporeSigma). Metabolites were compared to commercial standards (MilliporeSigma). Unlabeled carbon substrates, glucose (Research Products International) and glutamate (MilliporeSigma), were fed to yeasts. Stable isotopes, [1,2-^13^C_2_]glucose, [U-^13^C_6_]glucose, [U-^13^C_5_]glutamate, [U-^13^C_6_]mannose, [U-^13^C_6_]fructose, and [U-^13^C_2_]glycine, were obtained (Cambridge Isotope Laboratories, Tewksbury, MA, USA). General solvents used for biomass extraction (chloroform, hydrochloric acid, and isopropanol) and/or mass spectrometry analyses (acetonitrile, hexane, and methanol) were also acquired (Thermo Fisher Scientific). Ultrapure water for biomass extractions and liquid chromatography-tandem mass spectrometry (LC-MS/MS) analyses was from a Milli-Q system (MilliporeSigma).

### *H. capsulatum* strains and cultivation

The *Histoplasma* North American clade 2 (*H. ohiense*) clinical isolate G217B (ATCC 26032) was maintained in *Histoplasma*-macrophage medium (67) with continuous shaking (200 rpm at 37°C). For experiments in defined media, 3M medium with 80 mM glucose and 30 mM glutamate was inoculated at 5 million yeast/mL. The same media was also incubated without yeasts to control for evaporation. After 67 h of incubation, cultures were aliquoted to 250 million cells per tube, washed once with cold water, and devitalized in 500 µL of 60% methanol before freezing at −80°C.

### Biomass extraction and quantification

Samples were received at the University of North Texas, speed-vacuumed (30°C for 1 h), flash-frozen in liquid nitrogen, and lyophilized (−80°C for 3 days). Multiple extractions, each with five biological replicates, were performed to quantify fatty acids, glycogen, proteins, mannitol, and cell wall components from freeze-dried yeasts, while nucleic acids were extracted from live yeasts at The Ohio State University.

Fatty acids were extracted using methyl tert-butyl ether as described (68), and 50 µL of 1 mg/mL TG-C19:0 was added prior to homogenization as an internal standard (TG-C19:0 was below the limit of detection in the analysis of fatty acid methyl esters from *Histoplasma* [data not shown]). Fatty acid methyl ester quantification was performed using gas chromatography-mass spectrometry (GC-MS) (69) with an Agilent 6890N gas chromatograph coupled to an Agilent 5975B mass selective detector (Santa Clara, CA) and an Omegawax 250 capillary column (30 m × 0.25 mm × 0.25 µm; MilliporeSigma). Data acquisition and integration were performed as previously described with modifications: the initial oven temperature was 80°C and held for 0.5 min, followed by an increase to 245°C for 7.5 min at a rate of 25°C min^−1^ (70).

Protein and glycogen were sequentially extracted according to a hexane/isopropanol phase extraction method (71). Following lipid removal, the extracted protein underwent acid hydrolysis, and the resulting amino acids were analyzed by LC-MS/MS. Samples were analyzed using an Agilent 1290 Infinity II HPLC (Santa Clara, CA, USA) coupled to an ABSciex QTRAP6500+ mass spectrometer system (Framingham, MA, USA) following the described protocol (37). Hydrolyzed protein extract was resuspended in 0.5 mL hydrochloric acid (0.01 N) and then diluted 1:250 in 1 mM hydrochloric acid. Four microliters of diluted sample was injected onto the column. Total crude protein content was determined using a FlashSmart Elemental Analyzer (Thermo Fisher Scientific) through the Dumas combustion method at the Quantitative Bio-Element Analysis and Mapping Center at Michigan State University. Percent nitrogen was multiplied by the nitrogen-to-protein conversion factor, 6.25, to yield total crude protein (29). Glycogen was subsequently extracted by autoclaving in the presence of α-amyloglucosidase to release glucosyl units and quantified as described (72).

Mannitol was extracted and purified according to a boiling water extraction (16, 73–75). Two-hundred nanomoles of [U-^13^C_6_]mannose was added at the time of extraction as an internal standard. Lyophilized extracts were resuspended in 500 mL of ultrapure water, transferred to 0.2 µm NANOSEP microfilter tubes (Thermo Fisher Scientific), and centrifuged (17,000 × *g* at 4°C for 10 min). Purified extracts were diluted 1:500 in acetonitrile/water (70:30; vol/vol), and 2 mL was injected onto the column for LC-MS/MS analysis. Mannitol was quantified as reported (10).

For nucleic acids, 250 million yeasts were collected by centrifugation (2,000 × *g* for 2 min) and resuspended in 200 mL of lysis buffer (2% Triton X-100, 200 mM NaCl, 20 mM Tris, pH 8.0, and 2 mM EDTA). Approximately 100 µL of glass beads (0.5 µm diameter) and 200 µL of phenol:CHCl_3_:isoamyl alcohol (25:24:1) were added to the resuspension and agitated for 2 min. Lysate was clarified by centrifugation (14,000 × *g* for 10 min), and the aqueous (upper) nucleic acid-containing phase was diluted 1:4 in water. The nucleic acids were precipitated by adding 1/10 vol of 3M sodium acetate pH 5.2 and an equal volume of isopropanol for 5 min. The precipitated nucleic acids were collected by centrifugation (14,000 × *g* for 10 min), washed with 70% ethanol, resuspended in 50 mL TE (10 mM Tris, pH 8.0, 1 mM EDTA), and quantified by absorbance at 260 nm (WPA Biowave Spectrophotometer, Biochrom Ltd., Cambridge, England).

The monosaccharide composition of *Histoplasma*’s cell wall was determined as previously detailed through weak acid hydrolysis (2M trifluoroacetic acid), alditol acetate derivatization of extracts (requiring 10 mg/mL sodium borohydride in 1 M ammonium hydroxide, acetic anhydride, and pyridine), and GC-MS quantification of hemicellulose components (76). Amino sugars (chitin) were not compatible with the extraction and/or derivatization.

### Media consumption

Media consumption was assessed by detecting the remaining amount of substrates after incubation for 67 h. Four uninoculated media were incubated alongside four inoculated media to control for evaporation. Following incubation, 1 mL was collected from the inoculated or uninoculated media, and yeasts were removed by centrifugation (2,000 × *g* for 2 min) and filtered using Choice PVDF (Hydrophilic) Syringe Filters (Invitrogen). Ten microliters of media was diluted 1:100 with ultrapure water and 1 µmol of [U-^13^C_6_]fructose and [U-^13^C_2_]glycine were added as internal standards. Samples were cleaned using 0.2 µm NANOSEP microfiltering devices and quantified by LC-MS/MS as previously described (69, 71). Glucose and glutamate consumptions were determined by the difference between the substrate concentrations of uninoculated and inoculated media.

Total carbon uptake and CCE were calculated as described (35), where CCE is defined as the percentage of biomass accumulation versus total carbon uptake. To account for differences in the growth rates of the cultures, the average DW of aliquoted 250 million yeast samples was correlated to the inoculum (representing initial biomass) and final cell count (representing final biomass) to estimate biomass accumulation. The same calculation was applied to determine substrate consumption for the entire culture’s cell mass.

### Biomass rate calculation

Vwall, Vfas2, Vmaneff, Vpyrim, and Vpurine, defined in Table 1, were calculated from their respective biomass accumulation values (mg) using their monomer molecular weights minus the molecular weight from bonds involved in polymer formation and divided by incubation time and DW. Vfas2 was further determined by multiplying each fatty acid species (in nmol) by its respective number of acetyl-CoA monomers and summing their products. Calculation of the singular propionyl-CoA unit for odd-chain fatty acids was counted as 1.5 acetyl-CoA units. For Vfas1, Vfas2 was divided by three to account for the three fatty acids esterified to a single glycerol backbone in triacylglycerols and divided by the weighted average number of acetyl-CoA monomers per fatty acid species. For proteinogenic amino acid fluxes, individual amino acid weight was calculated from the total crude protein content to their individual percentage in the proteinogenic amino acid distribution. Then, weight was converted to nmol using the respective molecular weights for each amino acid minus peptide bond weight.

### Metabolite extraction and analyses

Water-soluble metabolites (sugars, sugar alcohols, amino acids, organic acids, and phosphorylated compounds) were extracted using boiling water from five biological replicates (four replicates for condition b), each containing 20 million yeasts, for each labeling condition: (i) unlabeled substrates, (ii) 20% [U-^13^C_6_]glucose + 20% [U-^13^C_5_]glutamate, (iii) 80% [1,2-^13^C_2_]glucose + 20% [U-^13^C_6_]glucose, and (iv) 100% [U-^13^C_5_]glutamate. LC-MS/MS analysis using multiple reaction monitoring was conducted on the different classes of metabolites and their respective mass isotopomer distributions as collectively reported (35).

### Calculating free fluxes

To estimate metabolic fluxes, a model of central metabolism was built and provided with the rates of substrate uptake in nmol h^−1^ mg DW^−1^ for glucose (Vglcup) and glutamate (Vgluup). Biomass fluxes were quantified according to the rate of DW and biomass accumulation for cell wall synthesis (Vwall), glycerol incorporation into triacylglycerols (Vfas1), fatty acid synthesis (Vfas2), mannitol synthesis (Vmaneff), synthesis of pyrimidines (Vpyrim), synthesis of purines (Vpurine), and synthesis of proteinogenic amino acids, with data from Fig. 1A through C. The label inputs consisting of either a mixture of 20% [U-^13^C_6_]glucose + 80% [1,2-^13^C_2_]glucose or 100% [U-^13^C_5_]glutamate and data from the parallel labeling experiments (Table S2) were also provided. The natural abundance of ^13^C was accounted for within INCA, and 0.5% was added to all isotopologue standard deviation percentages to avoid overfitting low-abundance isotopologues during flux estimation (77). Symmetrical mapping of atoms was included for fumarate, mannitol, succinate, and triose phosphates. Source fluxes (Vglcup and Vgluup) and sink fluxes toward biomass component accumulation (Vwall, Vfas1, Vfas2, Vmaneff, Vpyrim, and Vpurine) were included as experimental measurements in the model, with their respective standard deviations being used for flux estimation. Proteinogenic amino acid fluxes were constrained to their experimental average values. All other fluxes were estimated using the Levenberg-Marquardt optimization algorithm (78) by minimizing the variance-weighted sum of squared residuals between experimentally measured and simulated mass isotopomer distributions of intracellular metabolites. The ^13^C-enrichment data from experiments using ^13^C-glucose and ^13^C-glutamate were run simultaneously by selecting the “Run in parallel” function, and flux estimation was repeated 100 times from random initial values to ensure a global best fit. Monte Carlo analysis was employed to calculate the 95% confidence intervals for fitted fluxes, with 100 trials per iteration and an infinite number of trials possible. The relative error tolerance was set at 0.05.

## SUPPLEMENTAL MATERIAL

Fig. S1 msystems.00569-25-s0001.pdf Model-predicted labeling data accurately models experimental measurements.

Table S1 msystems.00569-25-s0002.xlsx Fractional labeling of intracellular metabolites in *Histoplasma* incubated with 20% [U-^13^C_6_]glucose + 20% [U-^13^C_5_]glutamate.

Table S2 msystems.00569-25-s0003.xlsx Steady-state ^13^C isotopomer abundance of intracellular metabolites incubated with 80% [1,2-^13^C_2_]glucose + 20% [U-^13^C_6_]glucose or 100% [U-^13^C_5_]glutamate.

Table S3 msystems.00569-25-s0004.xlsx Metabolic network detailing atom transitions.

ASM does not own the copyrights to Supplemental Material that may be linked to, or accessed through, an article. The authors have granted ASM a non-exclusive, world-wide license to publish the Supplemental Material files. Please contact the corresponding author directly for reuse.
