# Supplementary material for: Mapping carbon utilization pathways in Histoplasma capsulatum through 13C-metabolic flux analysis
Source: mSystems. 2025 Sep 8;10(10):e00569-25. doi: 10.1128/msystems.00569-25 (PMC12542744; doi:10.1128/msystems.00569-25)
Supplement: Fig. S1 — Model-predicted labeling data accurately models experimental measurements. [file msystems.00569-25-s0001.pdf]

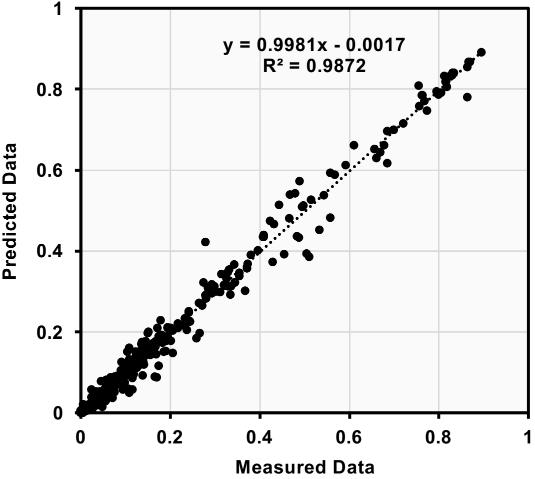

Fig. S1. Model-predicted labeling data accurately models experimental measurements. The linear regression equation and R^2 value are displayed.
